# Evaluation of universal newborn hearing screening in South African primary care

**DOI:** 10.4102/phcfm.v7i1.769

**Published:** 2015-05-21

**Authors:** Katijah Khoza-Shangase, Shannon Harbinson

**Affiliations:** 1 Faculty of Humanities, Department of Audiology, University of the Witwatersrand, South Africa

## Abstract

**Background:** Universal Newborn Hearing Screening (UNHC) is the gold standard toward early hearing detection and intervention, hence the importance of its deliberation within the South African context.

**Aim:** To determine the feasibility of screening in low-risk neonates, using Otoacoustic Emissions (OAEs), within the Midwife Obstetric Unit (MOU) three-day assessment clinic at a Community Health Centre (CHC), at various test times following birth.

**Method:** Within a quantitative, prospective design, 272 neonates were included. Case history interviews, otoscopic examinations and Distortion Product OAEs (DPOAEs) screening were conducted at two sessions (within six hours and approximately three days after birth). Data were analysed via descriptive statistics.

**Results:** Based on current staffing profile and practice, efficient and comprehensive screening is not successful within hours of birth, but is more so at the MOU three-day assessment clinic. Significantly higher numbers of infants were screened at session 2, with significantly less false-positive results. At session 1, only 38.1% of the neonates were screened, as opposed to more than 100% at session 2. Session 1 yielded an 82.1% rate of false positive findings, a rate that not only has important implications for the emotional well-being of the parents; but also for resource-stricken environments where expenditure has to be accounted for carefully.

**Conclusion:** Current findings highlight the importance of studying methodologies to ensure effective reach for hearing screening within the South African context. These findings argue for UNHS initiatives to include the MOU three-day assessment to ensure that a higher number of neonates are reached and confounding variables such as vernix have been eliminated.

## Abstrait

**Evaluation du test universel de dépistage auditif chez les nouveau-nés dans les soins primaires sud-africains.**

**Contexte:** Le Test universel de Dépistage auditif chez les Nouveau-nés (UNHC) est la norme d'excellence pour le dépistage et le traitement précoces de la surdité ; il est donc important d'en discuter dans le contexte sud-africain.

**Objectif:** Déterminer la faisabilité du dépistage chez les nouveau-nés à faible risque, au moyen d'Emissions otoacoustiques (OAE), au sein de la clinique d’évaluation de trois jours de l'Unité d'Obstétrique des Sages-femmes (MOU) dans un Centre de Santé communautaire (CHC), à différentes périodes d'essais après la naissance.

**Méthode:** On a inclus 272 nouveau-nés au sein d'une conception prospective et quantitative. On a effectué des entrevues sur les antécédents médicaux, des examens otoscopiques et des dépistages de produits de distorsion d’émissions otoacoustiques (DPOAEs) au cours de deux sessions (dans les six heures et environ trois jours après la naissance). Les données ont été analysées par satistiques descriptives.

**Résultats:** Selon le profil actuel et les pratiques du personnel, le dépistage complet et efficace n'est pas performant dans les heures suivant la naissance, mais il a plus de succès dans les cliniques d’évaluation de trois jours du MOU. Un plus grand nombre de bébés a été dépisté à la session 2, avec beaucoup moins de résultats faussement positifs. A la session 1, seuls 38.1% des nouveau-nés ont été dépistés, contre plus de 100% à la session 2. La session 1 a donné un taux de 82.1% de résultats faussement positifs, ce qui a des conséquences importantes pour le bien-être émotionnel des parents; mais aussi pour les environnements à ressources limitées où il faut rendre compte soigneusement des dépenses.

**Conclusion:** Les résultats actuels soulignent l'importance de l’étude des méthodologies pour assurer la portée efficace du dépistage de la surdité dans le contexte sud-africain. Ces résultats plaident pour que les initiatives de l'UNHS incluent l’évaluation de trois jours du MOU pour pouvoir inclure un plus grand nombre de nouveau-nés et que les facteurs de confusion comme le vernix soient éliminés.

## Introduction

The profession of audiology has focused on childhood hearing screening for several years and the screening for paediatric hearing impairment has subsequently become an important component of neonatal care. The Health Professions Council of South Africa (HPCSA) and the Joint Committee on Infant Hearing (JCIH) endorse, advocate and stipulate the early identification of hearing loss through the employment of objective physiological screening measures, so that the timely diagnosis and treatment for congenital auditory impairment may occur.^1,2^ This, therefore, highlights the intention of neonatal hearing screening, which is to ensure the early identification of congenital hearing impairment as well as the early intervention for those identified with a hearing loss.^3^ Screening for hearing impairment is viewed as a method of prevention and is mandated in several developed countries. Screening has also been deemed as an attainable public health programme in developing countries.^4^

The aim of neonatal hearing screening may be achievable through the appropriate screening of all infants,^5^ otherwise called universal newborn hearing screening (UNHS). UNHS refers to a prevention programme in which all newborns are screened for hearing impairment, after birth, prior to discharge from the newborn nursery.^6^ In contrast to UNHS, targeted hearing screening denotes a selective screening method based on the presence of established risk factors. According to Flynn *et**al.*^7^ a comparison between UNHS and targeted hearing screening procedures has indicated that universal hearing screening measures are generally preferred.

There is evidence to suggest that the lack of UNHS programmes may be detrimental to several hearing-impaired children. These newborn hearing screening programmes are considered to be valid and are thus likely to result in the timeous identification of, and intervention for, congenital hearing loss.^3^ The primary rationale underlying UNHS and the early detection of hearing impairment is that hearing-impaired children who are provided with suitable intervention services within the first six months of life, present with considerably better language skills when compared to children who receive this intervention at a later stage.^8^ Considering the age at which the detection of, and intervention for, hearing impairment occurs, a properly implemented neonatal hearing screening programme is able to offer acceptable outcomes in terms of language and emotional development, as well as educational and vocational outcomes.^9^ However, in the absence of an appropriate hearing screening programme, a hearing-impaired child may only be identified once the child is of school-going age.^3^ UNHS has therefore been proposed as a means to speeding up the identification, diagnostic and intervention process for hearing-impaired children.^10^ Neonatal hearing screening programmes are deemed as advantageous and are, therefore, accepted in many developed countries.^11^ These early hearing detection programmes have been implemented as components of the public health system in many countries^12^ and the establishment of UNHS programmes has been on the increase internationally.^3^

The increase in UNHS programmes may be a result of the existing evidence that UNHS is a cost-effective approach for the timeous and effective detection of congenital hearing impairment;^13^ and may also be attributed to reports of feasibility and value of such programmes.^4^ Neonatal hearing screening is gradually becoming a standard procedure internationally.^14^ However, it is of great concern that the implementation of extensive neonatal audiological screening drives has mainly been limited to the developed world. This implementation has not yet been intensified in the developing world, namely, the developing countries of Asia, Latin America, the Caribbean and Africa (and 80% of the world's population).^15^

If UNHS is valid, then it must also be established as effective and viable across geographically-varied hospital collections, with differing staffing attitudes and resources.^16^ This implies that UNHS needs to be embraced in the developing world, considering that most children with a hearing impairment are reported to live in third-world countries.^17^

According to Olusanya, Luxon and Wirz,^17^ the feasibility of newborn hearing screening programmes for developing countries seems inadequate in view of the diversities in the socioeconomic and health standing of these countries. This may be because of the perception of hearing impairment that, although hearing loss is debilitating, it is not life-threatening when compared to various fatal childhood diseases. In spite of this, a great number of developing countries are exploring practical and culturally-appropriate options for early hearing screening.

Whilst the available technology for newborn hearing screening is appropriate for employment in developing countries, the advantages and benefits of early detection and early intervention services for infants with a hearing impairment are not always available and easily reachable. Moreover, administrative systematisation for UNHS has not been established in several of these countries.^11^ Findings from ongoing infant hearing screening programmes in South Africa and in Nigeria have even proposed that hearing screening programmes be integrated into early childhood immunisation programmes in developing countries, especially where a number of births occur outside regular hospitals and clinic settings.^18^ However, regardless of the numerous recommendations,^19^ researchers have acknowledged that the establishment of a UNHS programme in settings with such limitations may be a challenging task.

Notwithstanding the challenges associated with the establishment and implementation of these UNHS programmes in developing countries such as South Africa, there are accessible structures that need to be explored and considered as potential platforms from which these programmes can be realised.^20^ In South Africa, the professional board for speech, language and hearing professionals has suggested that community-based developmental hearing screening programmes be put into operation at the primary healthcare level within the district health services model.^1^ Structures that may be explored include the community health centres (CHCs), where babies are followed up after being discharged from the hospital, hence the current study.

According to the JCIH,^2^ the establishment of suitable practices is a necessary part of the foundation for newborn hearing screening programmes. Intensified research and the development of appropriate screening programmes for the detection of, and intervention for, hearing impairment in the newborn population are required. There is a pressing call for further research comparing hearing screening programmes in various contexts; that is, research is required that aims to establish whether the programme, equipment and protocols are designed to meet the specific objectives according to the context.^21^ This is particularly true for developing countries, where resources are scarce and decisions are mostly financially driven.

Olusanya and Okolo^18^ have highlighted South Africa's means to realise valuable and feasible neonatal audiological screening programmes. However, in order to guide the implementation process of neonatal hearing screening programmes in South Africa, research to collate evidence concerning the efficacy and practicability of these screening programmes is required,^1^ hence the current study.

The primary aim of the study was to determine the feasibility of otoacoustic emissions (OAE) screening in low-risk neonates at different times and places following birth in a primary care setting. Specific objectives of the study were:

- To investigate the practicability and efficiency when OAE screening takes place within six hours after birth, prior to discharge from the newborn nursery.
- To investigate the practicability and efficiency when OAE screening takes place at three days after birth at the Midwife Obstetric Unit (MOU) three-day assessment clinic.
- To compare the findings of the OAE screening obtained across the two differing test times.

## Research methods and design

### Study design

This study employed a quantitative research design. Quantitative research designs entail the utilisation of standardised measures, with fixed categories, to which numbers are assigned. For the purposes of this study, the standardised measures were the audiological screening measures (otoscopic examinations and OAEs) and the fixed categories were the screening results obtained (*pass/refer*).^22^

Within the quantitative research design application for this study, a longitudinal approach was adopted. A longitudinal design, or within-subject design, involves the collection of data from the same sample of participants at two or more points in time.^22^ For the purpose of this study, two data collection sessions – one on the day of birth and then one at the Midwife Obstetric Unit (MOU) three-day assessment clinic, with approximately three days between the sessions – were adopted. All testing was conducted by a qualified registered audiologist.

### Setting

The study was conducted at a Community Health Centre's MOU department in Gauteng, South Africa. The CHC is run daily by midwives, with a majority of babies born there being discharged within six hours of birth, with a clinic follow up appointment for the MOU three-day assessment clinic. The CHC has an audiologist in its staff establishment who keeps 08:00–16:30 working hours on weekdays and attends every scheduled MOU assessment clinic. Dippenaar^23^ has described the South African context where midwives care for 77% of pregnant women and are, therefore, an integral part of the healthcare system. These midwives manage low-risk pregnancies, with high-risk pregnancy being referred to the hospital system. Hence, all the neonates attended to at the research site are considered to be low risk.^24^

### Study population

The target population for the current study was the low-risk neonatal population in Gauteng, whilst the accessible population was all neonates at the CHC where data were collected. All neonates during a one-month period were potentially included in the study (from 30 August to 30 September 2009), which had the following inclusion and exclusion criteria.^25^

Participants were required to be well, full-term neonates and to be born by normal vaginal delivery. A full-term neonate is one that is born at 38–42 weeks’ gestation.^26^ Participants were required to present with an unremarkable prenatal and perinatal history, as reported in the participant's clinic file.

Newborns older than seven days at session 2 were not included in this study. The rationale for this is that the aim of UNHS is to identify congenital hearing loss, a hearing loss present at birth.^27^ A postnatal hearing loss is a hearing loss which is acquired after the perinatal period,^28^ where the perinatal period refers to the period from 28 weeks’ complete gestation to day 7 after delivery.^29^ Based on this, the seven-day cut-off was applied in an attempt to differentiate postnatal hearing loss from congenital hearing loss.

Of the 272 participants, 149 (54.8%) were boys and 123 (45.2%) were girls. The mean age (SD) at session 1 was 4.2 (1.3) hours and at session 2 was 3.9 (1.1) days.

The sample for this study (*N*= 272) was further divided into three distinct groups: the first group (*n*= 99) comprised the neonates tested at session 1, the second group (*n*= 173) comprised newborns tested only at session 2 and the third group (*n*= 95) comprised neonates tested at both session 1 and session 2.

### Instrumentation and materials

The materials which were employed included a case history checklist form and a data collection form, a Heine mini 2000 otoscope and a GSI AUDIOscreener, as well as a sound level meter to monitor noise levels during the hearing screening. The GSI AUDIOscreener is a portable, hand-held screener with automatic operations for quick and simple screening and is designed for universal newborn hearing screening (UNHS) purposes.

Details pertaining to the pregnancy were included in the case history interview and were aimed at determining whether the pregnancy was a healthy one and whether any complications existed, as well as to determine the age of the mother, with younger than 15 years and older than 35 years being regarded as the limits for maternal age.^30^ Details regarding the birth, postnatal conditions and a family history of hearing impairment were also included in the case history, with the aim of establishing whether any risk factors were present. Although it did not form part of the set objectives of the current study, it was important to identify and document any possible risk factors for congenital hearing impairment which the participants presented with, as part of ensuring context-specific and context-relevant evidence.

### Ethical considerations

The current study was examined and approved by the appropriate ethics committee and has therefore been performed in accordance with the ethical standards laid down in the 2012–2013 World Medical Association's Declaration of Helsinki. Permission to conduct the study was also obtained from hospital management at the research site and the Head of Department of Speech Therapy and Audiology. Data collection only began following permission from the Human Research Ethics Committee (Medical) at the University of the Witwatersrand (Ethical Clearance Number: M090836).

### Test procedures

#### The collection of case history information

Following the attainment of informed consent for participation in the study, a case history was obtained. The case history information was drawn from the participant's clinic file and from interviews with the participant's mother. In the presence of a language barrier, the services of a trained interpreter were employed in order to ensure the gathering of adequate case history details and to facilitate clear communication between the researcher and the mothers of the participants.^31^

#### Audiological screening

Two newborn hearing screening test sessions occurred. The initial screening session (session 1) took place at the CHC in the MOU department's newborn nursery, within six hours of the participant's birth, before discharge from the birth facility. The second screening session (session 2) also took place at the CHC but as part of the scheduled MOU three-day assessment clinic, approximately three days after the participant's birth.

Tattersall and Young^32^ have suggested that, in the case of a healthy infant obtaining a *pass* result during the initial screening process, no additional testing is necessary. Despite this suggestion, for the purposes of this study, irrespective of the result obtained at the initial screening session, all neonates were booked for re-screening at the MOU three-day assessment clinic.

At each audiological screening session, an otoscopic examination was carried out in both ears on each participant. An otoscopic examination is a subjective procedure deemed to be useful in the assessment for the presence or absence of middle ear effusion and is used to examine the external auditory meatus in order to assist with the selection of an appropriate probe tip for further tests.^33^ In the past, specialists have purposely highlighted the diagnostic worth of the otoscopic examination, claiming that the appropriate utilisation of this procedure may lead to improved diagnosis of middle ear pathology.^34^ Although the otoscopic examination is a subjective measure, it is also a cost-effective and highly-rated diagnostic tool.^35^ The otoscopic examination, therefore, ought to form part of standard paediatric audiological evaluations.^36^

For the otoscopic examination, a *pass*result represented a clear external auditory meatus with no foreign bodies or debris in the external auditory canal, no obvious middle ear pathology and a visibly intact and healthy tympanic membrane. The otoscopic examination is a key stage in the newborn hearing screening process, as middle ear pathology and/or obstruction has a documented adverse effect on the detectability of OAE responses; and the presence of cerumen or vernix in the external auditory meatus is implicated frequently in failed hearing screenings.^37^

Following the otoscopic examination, a distortion product otoacoustic emissions (DPOAE) screening was conducted. In order to obtain a DPOAE response, a small probe is inserted into the participant's external auditory meatus.^32^ To enhance the probe fitting, the tester may remove and clean the probe tip and then re-test immediately^38^ and this was done from time to time in the current study.

The GSI AUDIOscreener was employed to obtain the DPOAE measures. This screener incorporates calibration within the test ear, which promotes total screening accuracy.^39^ The test parameters were set according to the default screening protocol setting – ‘Quick DPOAE’ – and three frequencies (2000 Hz, 3000 Hz and 4000 Hz) were assessed for each ear per participant. The criteria for an overall *pass*result were based on passing at least two of the three frequencies tested.

The hearing screening test results can be obtained in only a few seconds as DPOAE screening devices conveniently feature pass-fail algorithms.^3^ Screening methodologies that include automated response detection are preferable to the screening methodologies that require operator interpretation. Therefore, to decrease tester error, a programmed OAE machine with pass or fail criteria is recommended^40^ and was employed for the purposes of the current study. The audiological screening results obtained, per participant, across the two screening sessions, were recorded using the data collection form. The screening results were recorded across the *pass*/*refer* category. The term *refer* was used in place of the term *fail*, with the aim of emphasising that not passing the screening session indicates necessity for follow-up testing to confirm or exclude the presence of a hearing impairment.^41^

The overall *pass*criteria for the purposes of this research project were a normal otoscopic examination in both ears, as well as a bilateral *pass*result for the DPOAE screening. It has been suggested that newborns that do not pass the initial hearing screening session can be re-tested prior to hospital discharge.^42^ It has been implied that test repetition may result in a reduction in the high *refer* rates from UNHS.^13^ The overall specificity of a screening protocol can thus be increased by testing infants twice.^42^ In line with this, for the purposes of this study, participants not obtaining a *pass*result were re-screened immediately.

All neonates who do not pass the birth admission audiological screening session and any follow-up screening sessions are to undergo thorough audiologic and medical examinations in order to verify the presence of a hearing impairment before the infant is three months of age.^43^ Therefore, for the purposes of the current study, in the case of a neonate not passing the second screening session, the neonate was referred for a full audiological assessment.

OAE responses can be obtained in a non-soundproofed environment.^44^ Therefore, for the initial testing session, within six hours of birth, audiological screening took place in the post-delivery room, in the MOU department at the CHC. Screening was conducted whilst the neonate was lying in an open crib. The participants need not be asleep for the OAE testing, as an OAE can be obtained in various states of arousal.^45^ Screening during the second test session took place in the Rehabilitation department at the CHC, which is off the same corridor as the MOU department.

### Validity and reliability

A trained interpreter was employed when indicated in order to obtain an accurate case history for each participant. To ensure that an accurate case history was obtained, information obtained during the interview was cross-checked with the details recorded in the participant's clinic file. In terms of test procedures, the employment of an otoscopic examination, conducted prior to the OAE screening, ensured an accurate interpretation of the OAE result obtained and thus added to the aspects of validity and reliability. The OAE screening measure contributed to reliability and validity, as these screening measures are reportedly both reliable and sensitive.^46^ The appropriate screening equipment was utilised and protocol strictly adhered to across all participants. Protocols also remained constant between participants and calibration of the OAE machine was ensured. Furthermore, it has been suggested that, in order for OAE measures to be reliable, ambient noise levels should not exceed 50 to 55 dB A of noise.^47^ Noise levels in both of the test environments were measured using a sound level meter to ensure that the environment remained appropriate for audiological screening, that is between 50 to 55 dB A of noise.

### Data analysis and statistical procedures

This study entailed the collection of categorical data, used for classification purposes, where categorical data can be defined as the frequency of observations falling into various categories.^48^ The categories pertaining to this project were those of *pass*and *refer*.

In order to determine the feasibility of audiological screening in low-risk neonates, using OAEs, at different times following birth, various statistical tests were conducted. These included cross-tabulations and the matched pairs *t*-test.^49^

The number of neonates presenting with *refer* findings were analysed both unilaterally and bilaterally using descriptive statistics. After tabulation and coding of the data, followed by frequency distribution, measures of central tendency, variability, relative position and measures of relationship were adopted.

In terms of practicability, aspects taken into account included the availability of participants. Asma *et**al.*^50^ defines the coverage rate, which encompasses components of practicability, as the percentage born during the study that were tested, available resources in the form of staffing, the working hours of the audiologist and the time-frames of discharge from the newborn nursery, as well as the test equipment. In terms of efficiency, aspects taken into consideration included the results obtained for the otoscopic examination and for the otoacoustic emission, as well as the referral rate. The time taken per screening measure also forms part of the evaluation of the efficiency of OAE screening within six hours after birth; but this aspect was not measured in the current study. According to Hall,^51^ the OAE, when used as part of a UNHS programme, has a fairly short test time. The time taken to conduct the hearing screening on each participant is recognised as an aspect in the evaluation of the efficiency of OAE screening within six hours after birth, prior to discharge from the newborn nursery. However, this aspect was not formally measured as part of the current study, although it was noted from clinical experience on the part of the researcher audiologist that it was not problematic, so the time taken with each neonate was deemed appropriate for a screening session.

## Results

A sample of 272 low-risk neonates was screened for hearing impairment during the current study, across the two screening sessions. This sample comprised 149 male participants and 123 female participants.

### The practicability and efficiency of screening within six hours of birth

During the time of the current study, 260 neonates were born at the research site. However, only 99 (38.1%) of these newborns were screened at session 1. The 99 newborns screened at session 1 were available for screening at session 1; that is, the time period between the neonate's birth and discharge from the newborn nursery fell within normal working hours, when the audiologist was on duty to perform the screening. It is notable that the 99 newborns screened at session 1 comprised all the participants approached to participate in the study, as no participants declined the screening services as part of the current study. A total of 161 newborns were missed at the first screening session, as these neonates were born over weekends or during the night. The time period between these neonates’ births and their discharge from the newborn nursery did not fall within normal working hours when the audiologist was on duty to perform the screening.

In evaluating the efficiency of OAE screening at session 1; the screening results obtained have been taken into account. Of the 99 participants screened at session 1, 16 newborns obtained an overall *pass*result for the audiological screening and the remaining 83 participants obtained an overall *refer* result; which equates to an 83.8% refer rate. With the overall *pass*criteria for the purposes of the current study being a normal otoscopic examination bilaterally as well as a bilateral *pass*result for the DPOAE screening, the results for both the otoscopic examination as well as for the DPOAE screening at session 1 are detailed in Table 1 below.

**TABLE 1:** Summary of the screening results obtained during session 1 of the current study (*n* = 99 participants, 198 ears).

Procedure and result obtained	Unilateral	Bilateral
Otoscopic examination – Pass	7	17
Otoscopic examination – Refer	7	75
DPOAE – Pass	9	16
DPOAE – *Refer*	9	74
Total Participants examined	99	

DPOAE, Distortion Product Otoacoustic Emissions.

As depicted in Table 1, of the 99 participants screened at the first session; a small minority presented with *pass*findings, depicted by only 17 neonates presenting with a bilateral *pass*result for the otoscopic examination and 16 with a bilateral *pass* result for the DPOAE screening measure. A large majority presented with *refer*findings for both of these measures. Two newborns obtained a bilateral *pass*result for the otoscopic examination, yet obtained a unilateral *refer* result for the DPOAE screening. No neonates obtained a *refer* otoscopic examination result and a *pass* DPOAE screening result. These data are detailed in Table 2 below.

**TABLE 2:** Breakdown of screening results obtained during session 1 (*n* = 99).

Detailed results obtained	Number of participants
Number of bilateral *refer* results for Otoscopic examination and DPOAE screening.	74
Number of bilateral *pass* results for Otoscopic examination and DPOAE screening.	16
Number of unilateral *pass* results for Otoscopic examination and DPOAE screening on the left ear.	4
Number of unilateral *pass* results for Otoscopic examination and DPOAE screening on the right ear.	3
Number of bilateral *pass* results for Otoscopic examination, unilateral *refer* results for DPOAE screening.	2
Total number of newborns screened at session 1.	99

DPOAE, Distortion Product Otoacoustic Emissions.

### The practicability and efficiency of screening three days after birth

During the time of the current study, 260 neonates were born at the research site. It is noteworthy that a total of 268 neonates, 147 boys and 121 girls, were screened at the second screening session – 173 more than at session 1. This indicates that eight newborns not born at the CHC were also captured at the second screening session.

For session 2, there was still only one audiologist on duty from 08:00 to 16:30 on weekdays. As screening was conducted as part of the MOU three-day assessment programme, during scheduled times daily, no newborns were missed because of discharge time-frames or the audiologist's working hours.

In the evaluation of the efficiency of audiological screening at session 2, of the 268 participants screened at session 2, 266 participants obtained an overall *pass*result. Two participants obtained an overall *refer* result, which equates to an overall *refer* rate of 0.7% for the audiological screening results obtained during session 2. The audiological screening results obtained during the second screening session are detailed in Table 3 below.

**TABLE 3:** Summary of the screening results obtained during session 2 (*n* = 268).

Procedure and result obtained	Unilateral	Bilateral	Total participants examined
Otoscopic examination – *Pass*	1	267	268
Otoscopic examination – *Refer*	1	0	268
DPOAE – *Pass*	2	266	268
DPOAE – *Refer*	2	0	268

DPOAE, Distortion Product Otoacoustic Emissions.

There were no neonates that presented with bilateral *refer* results for both the otoscopic examination and the DPOAE screening measure during session 2. There was one participant that presented with a unilateral *pass*result for both the otoscopic examination and the DPOAE screening measure. In this case, the laterality of the ear in which the *pass*result for the otoscopic examination as well as the DPOAE result were obtained correlated. There was one newborn that obtained a bilateral *pass*result for the otoscopic examination, yet obtained a unilateral *refer* result for the DPOAE screening. It is worth noting that both the DPOAE *refer* results obtained were unilateral. There were no neonates that obtained *refer* otoscopic examination results and *pass* DPOAE screening results. This information is detailed in Table 4.

**TABLE 4:** Breakdown of screening results obtained during session 2 (*n* = 268).

Detailed results obtained	Number of participants
Bilateral *refer* results for Otoscopic examination and DPOAE screening.	0
Bilateral *pass* results for Otoscopic examination and DPOAE screening.	266
Unilateral *pass* results for Otoscopic examination and DPOAE on the left ear.	0
Unilateral *pass* results for Otoscopic examination and DPOAE on the right ear.	1
Bilateral *pass* results for otoscopic examination, unilateral *refer* results for DPOAE.	1
Total newborns screened at session 2.	268

DPOAE, Distortion Product Otoacoustic Emissions.

In terms of efficiency, the follow-up rate is to be taken into account. In the current study, of the 99 newborns that were screened at session 1, 95 participants returned for follow-up screening at session 2, as part of the MOU three-day assessment clinic. This equates to a follow-up return rate of 96%.

### Comparison of findings across the two screening sessions

During the time of the current study, 260 newborns were born at the research site. During session 1, only 99 newborns were screened, but 268 newborns were screened at session 2. The eight additional newborns who were screened at session 2 comprised babies born at home and not at the CHC during the same period, but whose parents still utilise the MOU three-day clinic for neonatal assessments and follow up, which is standard practice in the area.

A total of 161 neonates were missed at the first screening session as these neonates were born over weekends or during the night and, because of the discharge time-frames, the audiologist was not on duty to perform the screening. There were 268 newborns tested at session 2 as screening at session 2 was not affected by time of birth and discharge being outside of working hours. Audiological screening at session 2, as part of the MOU three-day assessment clinic, was the final screening session where referrals for diagnostic assessments were made.

In the current study, a total of 95 participants underwent screening at both sessions. The screening results for each of these participants has been captured across the two screening sessions and compared. It is notable that the majority of these participants (73.7%) obtained bilateral *refer* results at session 1 and then obtained bilateral *pass*results at session 2. There was one participant who obtained an overall *refer* result at both sessions and 16 participants who obtained an overall *pass* result at both sessions. A total of 78 participants obtained a *refer* result at session 1 and a *pass* result at session 2. It is notable that there were no participants that obtained a *pass* result at session 1 and a *refer* result at session 2.

It is also notable that there were no participants that obtained a *pass* result at session 1 that did not present for screening at session 2, but there were three participants with bilateral *refer* results at session 1 that did not present for re-screening at session 2.

In comparing the feasibility and efficiency of audiological screening at various times following birth, the number of *refer* results obtained across the two sessions has been taken into account. With regard to the otoscopic examination results, of the total *refer* results obtained, 99.4% of these were obtained during session 1; whilst only 0.6% were obtained at session 2. This indicates a considerably higher *refer* rate for otoscopic examinations at session 1 compared with session 2, approximately three days later.

In comparing the feasibility and efficiency of audiological screening at various times following birth, the number of DPOAE *refer* results obtained across the two sessions has been taken into account. Of the total *refer* results obtained; 98.8% of these were obtained during session 1, whilst only 1.2 % were obtained at session 2. The *refer* rate for DPOAE screening at session 1 is thus notably increased when compared with the rate at session 2.

It is notable that *p* < 0.0001, which indicates a very small chance that the differences are a result of variables other than group membership, where group membership refers to whether or not newborns were tested at session 2. The matched pairs *t*-test indicated statistically-significant differences between session 1 and session 2 *pass*/*refer*findings (*p*< 0.0001).^52^

## Discussion

The current study focused on low-risk newborns, as the newborns enrolled in the clinic system are considered to be low risk. Any newborns presenting with prenatal or perinatal conditions are referred to the hospital setting and would thus not be available for testing at the clinic or for participation in the current study. In light of this, noted risk factors for hearing impairment that were identified during the current study were collated and documented so that data could be analysed accordingly. In the current study, one newborn was identified as having a positive family history for permanent childhood hearing loss. Lahr and Rosenberg^53^ have listed this as a risk factor for hearing impairment. It is noteworthy that no other risk factors for hearing impairment were identified during the data collection of the current study. This is consistent with what would be considered appropriate in low-risk neonates and indicates that the current sample is representative of the general low-risk neonatal population.

During the time of the current study, 260 neonates were born at the CHC, yet only 99 of newborns underwent screening at session 1 – a mere 38.07%. There were no newborns whose parents refused screening at session 1; yet 161 newborns were missed at the first screening session.

**FIGURE 1:**
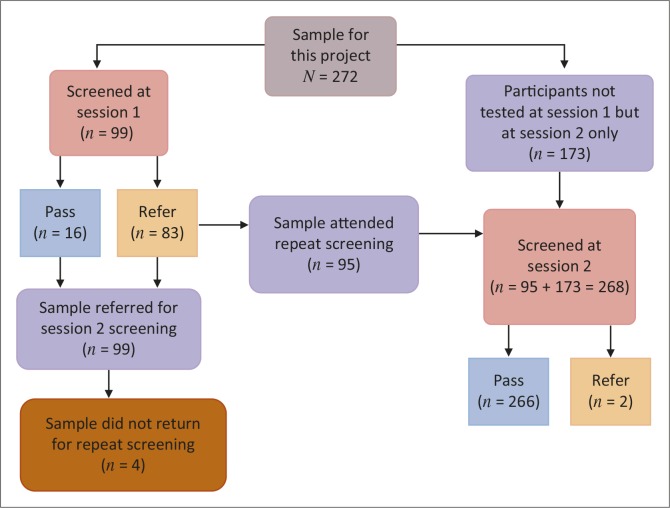
Summary of screening outcomes in the current study.

Factors contributing to the reduced number of participants at session 1 may include the time of birth, as the audiological screening at session 1 only took place at the centre during normal working hours, being on a Monday to Friday (08:00 to 16:30). Many newborns were born outside this time frame or were discharged within six hours of birth and were thus not screened. The findings from the current study are consistent with reports by Ng *et**al.*,^54^ where neonates were discharged without screening because of the time of birth and discharge outside of normal working hours. Another study by Abdullah *et**al.*^55^ reported that 10.8% of newborns were missed at the session 1 screening whilst, in the current study, 61.92% were missed at session 1. This number is significantly higher and has serious implications for the current context. The reasons for this, as documented by Abdullah *et**al.*,^55^ included discharge during weekends, absent screening personnel and neonates that were overlooked unintentionally. Although this is similar to the findings from the current study, a marked difference between the study by Abdullah *et**al.*^55^ and the current study exists. In the current study, newborns were discharged within six hours of birth; in the study by Abdullah *et**al.*,^55^ neonatal screening at session 1 took place within 24 hours of birth. The longer hospital stay meant that fewer newborns were missed because of working hour limitations in the study by Abdullah *et**al.*^55^ when compared with the current study. In spite of these findings, it is notable that Lim and Daniel^56^ have reported that screening prior to discharge after birth offers the greatest coverage. Nonetheless, this is a significant factor which reduces practicability of neonatal audiological screening using OAEs within six hours of birth in the context of the current study.

Adelola *et**al.*^57^ refer to a newborn hearing screening programme where the screening takes place in the maternity ward within 48 hours of birth from Monday through Friday. In their programme, the missed babies are sent for session 2 screening at the outpatient department. In a private healthcare setting, the minimal period for hospital stay post-birth is 24 hours and this is sufficient time to allow for universal newborn hearing screening to be conducted.^12^ In addition to this, it is possible for screening to be conducted from Monday through to Saturday in these contexts; again because of the availability of resources.^12^ This scenario is different to that in a government clinic where newborns are discharged from the clinic six hours post-birth and where the audiologist is only available to conduct the screening during normal working hours. This implies that the practicability for session 1 in this context is compromised. This has significant implications for implementing UNHS in CHC settings across South Africa, where similar protocols are followed in terms of discharge times and the availability of audiologists or other screening staff.

Early hearing detection and intervention (EHDI) coordinators are to be attentive to circumstances under which infants may be lost to the UNHS system. These may include home/out-of-hospital births and hospital missed screenings when infants are discharged prior to the hearing screening being conducted.^2^ This is especially significant for efficiency of screening at session 1 in the current study, where newborns are discharged within six hours of birth and where the audiologist conducting the screening is only available during normal working hours. Spivak^58^ has emphasised that a course for managing home births, early hospital discharge as well as private births needs to be instituted so that high coverage and consistent services can be delivered; and it is the opinion of the current researcher that this is crucial in a developing country such as South Africa.

In terms of resources related to staffing, there was only one audiologist on site to conduct the neonatal audiological screening. The audiologist adhered to normal working hours and this resulted in several newborns being missed at the initial screening session. This was proven to have a negative impact on the practicability of neonatal audiological screening at session 1. Theunissen and Swanepoel^59^ have stated that the most commonly reported grounds for the lack of neonatal screening programmes are the shortage of suitable screening equipment, as well as personnel shortages. Widen *et**al.*^41^ have also explained that trained nursing staff and volunteers are able to conduct newborn hearing screening tests, which is consistent with the statement made by Hayes^60^ that newborn hearing screening can be conducted by trained volunteers. Hayes^60^ has, however, stipulated that an audiologist's supervision is required in this event. The notion of newborn hearing screening being conducted by non-audiological staff is supported by the study conducted by Ferro *et**al.*,^61^ where Newborn Hearing Screening programmes in Illinois were compared. In their study, hearing screening was conducted most commonly by the nursing staff.

Throughout areas such as Latin America, the availability of hearing healthcare professionals is limited, especially in rural communities.^62^ In the study by Chan and Leung,^63^ the screening was conducted by enrolled nurses who had received training on OAE testing. These nurses conducted automated OAE screening and performed standard nursing duties as well. In contrast to this, in the current study, screening was only carried out by a qualified audiologist. This is the standard protocol in South Africa, for the most part; and it was also a result of time and resource limitations. Chan and Leung^63^ report that UNHS programmes, where screening is conducted by nurses, is a practical option; with concentrated and direct training. In the current study, in the event of the audiologist being ill for a day, the programme would be gravely affected as no other screening staff were available; a finding that can be generalised to a majority of clinics in South Africa as similar staffing and scope of practice conditions apply.

Hall^51^ has stated that universal newborn hearing screening through the use of OAE measures can be recorded dependably by non-audiologic personnel. In the current study, if screening was conducted by trained nursing staff, this would have meant that screening could have been conducted seven days a week and 24 hours a day. Thus the number of newborns missed at session 1 would have been greatly reduced.

In determining the efficiency of screening at session 1 for the current study, the audiological screening results, an overall *refer* rate of 83.83% was obtained. In light of such a high *refer* rate, it is essential to consider the possibility of false-positive results where a neonate does not pass the hearing screening but does not truly present with a hearing impairment.^64^ In the case of neonatal audiological screening, false-positive screening results have been reported as being a major concern.^16^ False-positive results may be obtained when the transmission of sound from the earphone to the cochlea and back to the recording microphone is interrupted.^65^ Screening newborns on the day of birth is of particular concern because of the presence of vernix in the external auditory meatus.^65^ Based on this fact, the high *refer* rate at this screening session is not unexpected. Albuquerque and Kemp^66^ have stated that, when newborns are discharged from the birthing facility within six hours of birth, OAEs will render an unacceptably high false-positive rate; a finding supported by the results of the current study. Hall^51^ has stated that the higher the *refer* rate is, the lower the OAE specificity is; and this, therefore, has a negative impact on the efficiency of screening at session 1 for the current study. This does highlight the pitfalls of screening at this time and reduces the efficiency of screening at this session. These findings are not consistent with the American Academy of Paediatrics,^67^ where it has been stated that OAEs render a 5% – 20% *refer* rate in the first 24 hours post-birth. The findings in the current study are also not consistent with the results reported by Abdullah *et**al.*,^55^ where, at session 1 within 24 hours of birth, a *refer* rate of 19.7% was obtained. The reason for this inconsistency may be attributable to the time difference, namely, six hours for the current study and the 24 hour discharge time-frame for the study by Abdullah *et**al.*^55^ Current study findings provide evidence that, within the South African context, screening prior to discharge (which is often within six hours of birth) might not be the best time and might also be more detrimental than beneficial because of the impact of false-positive findings on the mother's well-being.

The second objective of the current study was to determine the practicability and efficiency when OAE screening takes place approximately three days after birth as part of the MOU three-day assessment clinic. The larger number of infants covered in session 2 implies that newborns born outside the CHC presented to the clinic and were included at the second screening session. Place of birth may influence the outcomes of a UNHS programme as this has an impact on the number of newborns that cannot be tested at session 1 – purely because they may not have been born at the CHC. Olusanya and Somefun^68^ have emphasised that a sizeable percentage of neonates with hearing impairment in numerous developing countries are born outside hospital settings. This accentuates the necessity for community-oriented UNHS, which will lead to early detection and intervention. In terms of coverage, Akhtar *et**al.*^69^ have stated that, in order to identify all newborns with a hearing loss, all newborns need to be screened. In developing countries, many newborns with sensorineural hearing loss are born at home and, therefore, session 2 testing may be more practical as these newborns can then be included in the screen as well.^68^ Based hereon, it is evident that screening newborns for hearing loss at the MOU three-day assessment clinic is practicable, as more newborns can be tested during this time-frame.

For the purposes of the current study, only one hearing healthcare professional was on duty at CHC. However, the impact of this staffing limitation was less influential at the second screening session as newborn screening was only conducted during scheduled times of the day and there was thus no impact resulting from discharge time and birth times. The MOU three-day assessment clinic is where medical check-ups on both the mother and baby take place, so the attendance is higher since the neonate will be undergoing a medical examination as well as a hearing screening. This highlights the importance of scheduling a hearing screening at the same time as a routine medical check-up. This will ensure that attendance is less costly for the parents in that it is cost effective to come for a single appointment to see several professionals than to present for appointments on different days. Ng *et**al.*^54^ have stated that the ideal time for screening would be when the neonate and mother present for a routine medical check-up. The findings of the current study support this, which again highlights the value of the MOU three-day assessment clinic, where both the mother and child present for a post-birth medical check-up.

Bartley and Digby^70^ have reported that OAEs stabilise after day 2 post-birth and this may explain the decrease in the number of *refer* results obtained at the second screening session in the current study. In a study conducted by Vaid *et**al.*,^71^ 1238 well newborns were screened. In their study, a *refer* rate of 11.14% was reported when newborn hearing screening was conducted at three days post-birth. This finding is consistent with the results reported by Doyle *et**al.*,^72^ where 200 well newborns were tested at five to 120 hours post-birth. These authors have reported that the OAE *pass* rate increases in infants older than 24 hours. The findings of the current study are consistent with this as a *refer* rate of 0.74% was obtained at session 2. The high *refer*rate at session one reduces the efficiency of session 1 as a platform for UNHS. The JCIH^2^ has stated that less than 4% of newborns should fail audiological screening at session 1 and at session 2 before being referred for diagnostic tests. The HPCSA stipulates that a referral rate of less than 5% should be achieved.

In the current study, session 1 does not meet the stipulated criteria and this implies that session 1 may not be a feasible test time; and might actually be a costly exercise in an already resource-stricken environment.

In terms of efficiency, the follow-up return rate was taken into account. In the current study, 95 of the 99 neonates screened at session 1, returned for follow-up screening at session 2. This equates to a follow-up return rate of 95.95% and indicates that session 2 is efficient as a platform for UNHS. This return rate is significantly better than the HPCSA benchmark of a minimum rate of 95%. The HPCSA stipulates that a 70% or greater follow-up return rate of infants and their caregivers is ideal.

Abdullah *et**al.*^55^ highlights the fact that audiological screening before 24 hours of age does result in a high false-positive rate. Consistent with the findings obtained in the current study, Stevens and Parker^73^ have outlined how the *pass*rate for OAE neonatal screening is reduced in the first 24 hours after birth. It has also been stipulated by Wada *et**al.*^11^ that the accuracy of newborn hearing screening seems to improve with time. This highlights the value and reliability of screening at a time outside the first 48 hours post-birth, when vernix no longer has an impact on the findings and at a time when parents are still eager to return to the clinic for follow-up visits.

In agreement with this, Torrico *et**al.*^74^ have suggested that screening should not take place within the first 24 hours of birth. Sun *et**al.*^75^ conducted a study in which various time intervals for in-patient UNHS were compared and results have indicated that testing on day 3 was more effective than screening on the first or second day post-birth. These findings and notions are in line with the findings of the current study.

The results of the current study are similar to the results obtained from the research conducted in Sweden by Hergils.^76^ In that study, 14 287 newborns at two maternity wards were screened over two sessions. Session 1 took place on the day of birth and session 2 took place at three days post-birth. The results of their study indicate that screening on the day of birth is less effective than screening on day 2 or 3 after birth.^76^ This is consistent with Gabbard, Northern and Yoshinaga-Itano,^77^ who have also reported a significant difference in OAE screening within the first 24 hours after birth and thereafter.

## Conclusion

Research strives to contribute to a scientific body of knowledge and aims to enhance health services and health outcomes. In the current study, a community-based newborn hearing screening programme has been considered in terms of efficacy and practicability. Research in this field is important as the drive behind the execution of extensive neonatal hearing screening programmes has not yet reached developing countries where more than half of the world's hearing impaired children reside. Current findings indicate that a need exists for the establishment of community-oriented primary ear care services in the developing world.

The current research project has addressed one of the many barriers regarding newborn hearing screening – that of the timing of neonatal OAE audiological screening, relative to post-birth discharge. The researcher has thus strived to ascertain the impact that time frames for neonatal audiological screening may have on the dependability of these programmes in primary healthcare settings in South Africa. Through the current study, the practicability and efficiency of an audiological screening programme within the MOU three-day assessment clinic has been positively proven.

The HPCSA^1^ position statement on hearing screening has referred to three hearing screening contexts: the well-baby nursery, on discharge from the neonatal intensive care unit, as well as Mother Child Health Clinics at the six-week immunisation clinic. The current study has rendered results that suggest an additional screening platform not previously considered or recommended. Whilst the HPCSA has made bold and positive recommendations and has proposed guidelines regarding EHDI, contextualising such recommendations remains crucial. Current findings have verified that the MOU three-day assessment clinic could be one of the most appropriate test times and may present as a suitable platform to roll out neonatal audiological screening in South Africa. This platform would ensure wide coverage, whilst keeping the rate of false-positive test results at a minimum.

Current findings have also emphasised the importance of having personnel other than an audiologist conducting the hearing screening. This would ensure that, if UNHS had to be conducted before discharge, personnel such as nurses or midwives who are available 24 hours every day could conduct the screening.

The outcomes of the current study add to the development of methodologies for the early identification of hearing impairment within the South African neonatal population.
